# 

**Published:** 1916-10

**Authors:** 


### CONTENTS.

ORIGINAL COMMUNICATIONS—

Gastro-Colic Fistula due to Chronic Gastric. The Enema an humble but Invaluable Agent.—By George M.M. Niles, M. D., Gastroenterologist to Georgia Boptist Hospital; consulting Gastroenterologist to the Anti-Tuberculogist Assodiation, etc. Atlanta, Ga. ........ 293

Ulcer Spontaneous Cure Case Report—by W. C. Gewin, M. D., Birmingham, Ala. ........ 304

Surgery and the Surgeon.—Ey Dr. Alfred Brown, Candler Building, Atlanta, Ga. ........ 307

Silvol in Gonorrhea. — By E. P. Merritt, M. D., Atlanta, Ga. Instructor in Genito-Urinery Surgery, Atlanta Medical College; Medical Department of Emory University. Urologist to Atlanta Anti-Tuberculosis Association. ........ 212

EDITORIAL ........ 315

SELECTIONS AND ABSTRACTS ........ 317

